# Editorial: Pharmacological therapy in patients with arrhythmias

**DOI:** 10.3389/fphar.2024.1465539

**Published:** 2024-07-25

**Authors:** L. Sciarra, S. Romano, G. Paparella, A. Scarà

**Affiliations:** ^1^ Cardiovascular Disease Department, University of Study L’Aquila, L’Aquila, Italy; ^2^ Clinique de L’Europe, Uccle, Belgium; ^3^ San Carlo di Nancy-GVM Hospital, Rome, Italy

**Keywords:** arrhythmias, pharmacological traetment, atrial fibrillation, DOAC, direct-acting oral anticoagulant, ischaemic stroke

Cardiac arrhythmias represent one of the most worldwide health problems. The incidence and prevalence of the different forms of arrhythmia can be significantly different in relation to age group and gender (Brugada et al., 2020): for example, the supraventricular tachycardia (SVT) prevalence is 2.25/1,000 persons and the incidence is 35/100,000 person-years. Women have a risk of developing SVT that is two times greater than men, and people aged >65 years or have more than five times the risk of developing SVT than younger individuals. On the other hand, WPW syndrome can be more frequently diagnosed in paediatric and young adult populations (Orejarena et al., 1998).

Atrial fibrillation is the most frequent cardiac sustained arrhythmia. The currently estimated prevalence of AF in adults is between 2% and 4% (Benjamin et al., 2019) and a 2.3 fold rise is expected (Colilla et al., 2013; Krijthe et al., 2013; Chugh et al., 2014). Increasing age is a prominent AF risk factor. Moreover, increasing burden of other comorbidities (hypertension, diabetes mellitus, heart failure, coronary artery disease, obesity and obstructive sleep apnoea) can play a crucial role (Cadby et al., 2015; Hobbelt et al., 2017; Aune et al., 2018; Grieco et al., 2018). AF is associated with substantial morbidity and mortality: in fact, AF can be recognized as cause of 20%–30% of all ischaemic strokes (and 10% of cryptogenic ones), can lead to left ventricular dysfunction and heart failure, consistently impairing quality of life patients and in some cases increasing incidence of death (1.5–3.5 fold rise) (Brugada et al., 2020).

For these reasons, early detection of AF represents an important goal; for this purpose, different tools of ECG monitoring (prolonged Holter ECG records, external loop recorder, event recorder, implantable devices) can be useful (de Ruvo et al., 2016; Sciarra et al., 2022).

In addition, maintenance of sinus rhythm can lead to better clinical outcomes, in patients suffering from AF: quality of life improves in symptomatic patients and cardiac mortality appears reduced in the setting of heart failure (Crawford et al., 2024). To achieve this objective, the most powerful weapon available seems to be catheter ablation. However, despite the different kind of energy useable and the enormous amount of techniques developed to treat this arrhythmia, ablation procedure appears far from to be considered a definitive solution (Sciarra et al., 2014; Scarà et al., 2018; Sciarra et al., 2018; Rebecchi et al., 2021; Sciarra and Scarà, 2023), even if a tailored approach can allow increasing efficacy, minimizing procedural risks (Palamà et al., 2022; Palamà et al., 2023).

Additional pharmacological therapy, to maintain sinus rhythm after cardiac ablation, can be a helpful tool as suggested by Fei et al. in this Research Topic: they presented a retrospective observational study that encompassed 420 elderly patients with AF following ablation procedure. Predictors of early recurrences were identified in left atrial size, left ventricular dimensions and some humoral biomarkers; in this setting, compared to amiodarone, propafenone and sotalol exhibited an elevated risk of early recurrence.

Prevention of ischaemic stroke is another crucial point in patients affected by AF. This Research Topic is extensively covered in this Research Topic: Yin et al. have drawn up a vademecum of 17 recommendations on the use of fondaparinux in numerous settings, including that concerning the treatment of patients after a cardioversion for atrial fibrillation. An important part of this Research Topic is dedicated to direct-acting oral anticoagulants (DOACs), suggesting, in some cases, particular usage: Pan et al., for example, explored the feasibility and safety of twice daily rivaroxaban as a postoperative anticoagulation regimen for patients with AF undergoing left atrial appendage closure (LAAC). They found that a short course of twice-daily rivaroxaban following LAAC is a feasible alternative regimen with a low rate of major bleeding events, and device-related thrombosis (DRT), thromboembolic events for patients with AF.

Furthermore, usage of DOACs, could open new scenarios for the management of anticoagulation in sports players affected by AF. Minardi et al., for example, suggested that the pharmacological profile of DOACs could offer theoretical solutions for overcoming the increased risk of bleeding in sports patients: drugs with lower half-lives could allow sports at risk of trauma within the “therapeutic window,” when the estimated blood concentration of the drug is minimal.

Finally, present Research Topic underline the importance of a rigorous management of anticoagulation therapy to reduce the incidence of haemorrhagic events in AF patients: Zhang et al. showed the beneficial effects of a national centralized drug procurement policy on anticoagulation selection in terms of medical adherence and reduction of adverse events.
